# Phylogeny and species delimitations in the entomopathogenic genus *Beauveria* (Hypocreales, Ascomycota), including the description of *B. peruviensis* sp. nov.

**DOI:** 10.3897/mycokeys.58.35764

**Published:** 2019-09-09

**Authors:** Danilo E. Bustamante, Manuel Oliva, Santos Leiva, Jani E. Mendoza, Leidy Bobadilla, Geysen Angulo, Martha S. Calderon

**Affiliations:** 1 Instituto de Investigación para el Desarrollo Sustentable de Ceja de Selva (INDES-CES), Universidad Nacional Toribio Rodríguez de Mendoza, Chachapoyas, Amazonas, Peru Universidad Nacional Toribio Rodríguez de Mendoza Chachapoyas Peru; 2 Laboratorio de Biología Molecular, Universidad Nacional Toribio Rodríguez de Mendoza, Chachapoyas, Amazonas, Peru Universidad Nacional Toribio Rodríguez de Mendoza Chachapoyas Peru

**Keywords:** *
Beauveria
*, fungal diversity, multi-locus phylogeny, Peru, polyphasic approaches, species delimitation

## Abstract

The genus *Beauveria* is considered a cosmopolitan anamorphic and teleomorphic genus of soilborne necrotrophic arthropod-pathogenic fungi that includes ecologically and economically important species. Species identification in *Beauveria* is difficult because of its structural simplicity and the lack of distinctive phenotypic variation. Therefore, the use of multi-locus sequence data is essential to establish robust species boundaries in addition to DNA-based species delimitation methods using genetic distance, coalescent, and genealogical concordance approaches (polyphasic approaches). In this regard, our study used multilocus phylogeny and five DNA-based methods to delimit species in *Beauveria* using three molecular makers. These polyphasic analyses allowed for the delimitation of 20–28 species in *Beauveria*, confirming cryptic diversity in five species (i.e. *B. amorpha*, *B. bassiana*, *B. diapheromeriphila*, and *B. pseudobassiana*) and supporting the description of *B. peruviensis* as a new taxon from northeastern Peru. The other five species were not evaluated as they did not have enough data (i.e. *B. araneola*, *B. gryllotalpidicola*, *B. loeiensis*, *B. medogensis*, and *B. rudraprayagi*). Our results demonstrate that the congruence among different methods in a polyphasic approach (e.g. genetic distance and coalescence methods) is more likely to show reliably supported species boundaries. Among the methods applied in this study, genetic distance, coalescent approaches, and multilocus phylogeny are crucial when establishing species boundaries in *Beauveria*.

## Introduction

Around 1800, a silkworm disease called “calcine”, “real del segno” or “muscardine” was causing great trouble in Italy and France (Redaelli and Visocchi 1940). Experiments developed by Agostino Bassi in Mariago, Italy showed that a parasitic fungus produced this disease (Redaelli and Visocchi 1940). Balsamo (1835) confirmed this discovery and concluded that the incrustation and white efflorescence, which covered the body of a dead silkworm, were a fungus of the genus *Botrytis*. He first named this species *Botrytis paradoxa* Balsamo and later *Botrytis bassiana* Balsamo (Balsamo 1835). Then, this species was transferred to its own genus and *Beauveria* Vuillemin was established on the basis of *B. bassiana* Vuillemin as the type species (Vuillemin 1912).

The genus *Beauveria* is considered a cosmopolitan genus of soilborne necrotrophic arthropod-pathogenic fungi that includes ecologically and economically important species (Rehner et al. 2011, Kepler et al. 2017, Chen et al. 2018). Morphologically, *Beauveria* genus have been characterized asexually by having conidiogenous cells arising from short, often one-celled, more or less swollen stalk cells, often in dense clusters, or scattered or in whorls from undifferentiated hyphae; they consist of a globose to fusiform basal part, and a geniculate, denticulate rachis. Conidia one-celled, hyaline, smooth, thin-walled, globose to ellipsoidal (de Hoog 1972). The sexual morphs form stromata solitary, paired or gregarious, unbranched, fleshy texture, fertile area apical, cylindrical to clavate, yellowish to orange; perithecia partially immersed, in longitudinal section oval to ovoid; and asci hyaline with cylindrical and filiform ascospores (Kepler et al. 2017).

Based on the end of dual nomenclature for different morphs of the same fungus in 2011 (McNeill et al. 2012), Kepler et al. (2017) phylogenetically established the genetic boundaries in *Cordycipitaceae* regardless of life-stage or the associated morphological differences. One of the most significant changes was the recognition of *Beauveria* as a genus separate from *Cordyceps*. Although direct links between species of *Beauveria* and cordyceps-like sexual morphs have been demonstrated from molecular data and culture-based experiments (Shimazu et al. 1988, Li et al. 2001, Huang et al. 2002, Shrestha et al. 2014), their respective type species are not congeneric (Kepler et al. 2017). Thereby, the clade composed of *Beauveria* currently includes the traditional species known from asexual morphs, as well as several taxa previously described for sexual morphs in *Cordyceps* (Sanjuan et al. 2014, Kepler et al. 2017).

Initially, *Beauveria* was delimitated based on diagnostic features, and three species were recognized, i.e., *B. bassiana*, *B. brongniartii* and *B. alba* (Limber) Saccas (de Hoog, 1972). New additions were included by de Hoog and Rao (1975), Samson and Evans (1982), Bissett and Widden (1986) and Rehner et al. (2006). Molecular analyses confirmed the monophyly and placement of seven species of *Beauveria* within Cordycipitaceae (Rehner and Buckley 2005, Sung et al. 2007). More recent molecular studies based on multilocus phylogenetic analysis that included the *Bloc* nuclear intergenic region, internal transcribed spacer (ITS), translation elongation factor-1α (*TEF*), and RNA polymerase II largest subunit (*RPB*1) and second largest subunit (*RPB*2) demonstrated that *Beauveria* is composed of 26 species (Rehner et al. 2011, Sanjuan et al. 2014, Kepler et al. 2017, Chen et al. 2018). These phylogenetic studies also revealed that the most commonly reported species, namely, *B. bassiana* and *B. brongniartii*, encompass cryptic lineages with worldwide distributions (Rehner et al. 2006, 2011, Ghikas et al. 2010). Although morphologically distinctive as a genus, species identification in *Beauveria*, especially in the conidiogenic state, is difficult because of its structural simplicity and lack of distinctive phenotypic variation. Thus, numerous registered mycoinsecticide formulations based on *B. bassiana* and *B. brongniartii* that are extensively used for the control of insect pests worldwide (Faria and Wraight 2007) are not likely based on these species (Rehner et al. 2006).

In the Amazonian region, a total of five species have been reported (Rehner et al. 2011, Sanjuan et al. 2014). Two of these species *B. acridophila* (T. Sanjuan & Franco-Mol.) T. Sanjuan, B. Shrestha, Kepler & Spatafora and *B. diapheromeriphila* (T. Sanjuan & S. Restrepo) T. Sanjuan, B. Shrestha, Kepler & Spatafora, and a lectotype, namely, *B. locustiphila* (Henn.) B. Shrestha, Kepler & Spatafora were recently described on the basis of molecular data and their sexual stages were characterized (Sanjuan et al. 2014). Additionally, two species of *Beauveria* were reported from Peru: *B. amorpha* Samson & Evans and *B. bassiana*, but only the former has been confirmed by molecular analysis while the latter is extensively used in coffee rust programs to control the expansion of the coffee borer (Rehner et al. 2011).

Given the problems with species delimitation in fungi using morphology, molecular data are becoming the standard for delimiting species and testing their traditional boundaries (Rehner et al. 2011). The recognition of distinct clades in gene trees as species is likely to be misleading in understanding the evolutionary history of taxa (Liu et al. 2016). Therefore, the use of multi-locus sequence data is essential to establish robust species boundaries (Lumbsch and Leavitt 2011). Most researchers, however, did not carefully examine the species boundaries but simply recognized distinct clades in single-gene trees as separate species (Stewart et al. 2014). Estimating the species tree and species delimitation using genetic distance (e.g. automated barcode gap discovery algorithm, ABGD; and statistical parsimony, SPN), coalescent (e.g. generalized mixed Yule coalescent, GMYC; and Bayesian phylogenetics and phylogeography, BPP), and genealogical concordance (genealogical concordance phylogenetic species recognition, GCPSR) methods have proven very useful and have been used for a range of animal and plant taxa (Liu et al. 2016). These methods have otherwise not been used much in fungi, especially in studies of pathogenic fungi (Millanes et al. 2014, Liu et al. 2015). Therefore, the use of several methodologies and data sets to delimit species is recommended, and subsequently, the achievement of congruent results across the methods is likely to prove most useful for framing reliably supported species boundaries (Carstens et al. 2013).

In this study, we analyzed species of the newly circumscribed genus *Beauveria*, including an unreported species isolated from coffee farms in northeastern Peru, based on morphological observations, phylogenetic inferences, and DNA-species delimitation methods. Three nuclear molecular markers (*Bloc*, *rpb*1, and *tef*1) were used to examine their phylogenetic relationships and to assess species boundaries within the genus *Beauveria*.

## Materials and methods

### Collection of specimens and isolation

**Figure 1.**
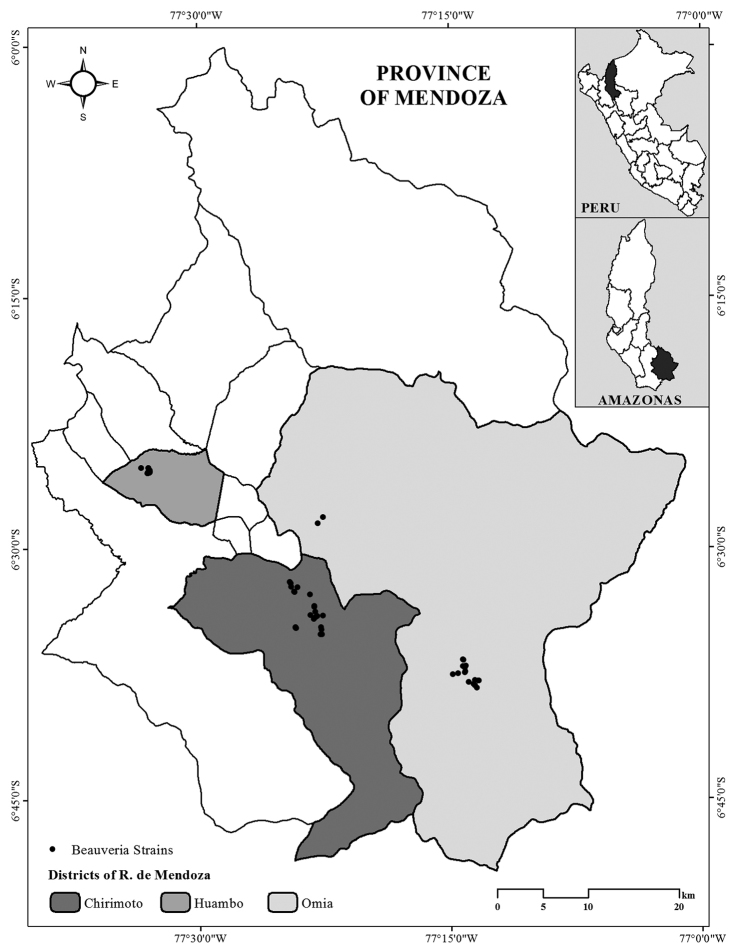
Collections of the 55 strains of *B. amazonensis* sp. nov. from the Rodriguez de Mendoza Province.

Fungal strains were isolated from infected coffee borers (*Hypothenemus hampei*) obtained from infected coffee berries according to Gerónimo-Torres et al. (2016). They were collected during July and August 2017 from three districts in the province of Rodriguez de Mendoza, Amazonas, Peru (Fig. 1). Briefly, infected coffee berries were preserved at 5 °C until coffee borers were recovered from them. The coffee borers with signs of fungal infection were cleaned superficially in 0.5% sodium hypochlorite solution and rinsed with sterile distilled water. Then, insects were placed in a humid chamber (90% RH and 25 °C) for 8 days to allow the growth of the entomopathogenic fungus. Once visible mycelia appeared on the borers under observations with a stereo microscope (Nikon SMZ18, Tokyo, Japan), these were transferred to a Petri-dish containing potato dextrose agar (PDA; Merck, Darmstadt, Germany).

### Identification of isolates

Fifty-five fungal strains were incubated as monosporic cultures on PDA at 25 °C for 15 days. Morphological characterization of the fungus was performed as described by Rehner and Buckley (2005). Microscope observations were made from fungal mycelia and other structures stained with methylene blue (0.1–0.5%). Photomicrographs were taken under an inverted microscope (IX83; Olympus, Tokyo, Japan) with an integrated camera (Nikon D810, Tokyo, Japan). Fungal strains were deposited as semisolid and dry material in the herbarium of Toribio Rodriguez de Mendoza National University (UT), Peru.

### Molecular phylogenetic analyses

Genomic DNA was extracted from semisolid PDA cultures using the NucleoSpin Plant II Kit (Macherey-Nagel, Düren, Germany), following the manufacturer’s instructions. Three genes were sequenced, i.e., *Bloc*, *rpb1*, and *tef1*. Each gene was amplified using polymerase chain reaction (PCR) with MasterMix (Promega, Wisconsin, USA) in the following reaction mixture: 10 ng of DNA and 0.25–0.5 pmol of forward and reverse primers for a total volume of 10 μl. The PCR protocols and primer combinations for *Bloc* (B5.1F, B5.4F, B3.1R, B3.3R), *rpb1* (RPB1A, RPB1A_VH6R, RPB1B_VH6Fa, RPB1B_G2R), and *tef1* (983F, 1567RintB) followed Rehner et al. (2011). The sequences of the forward and reverse strands were determined commercially by Macrogen Inc. (Macrogen, Seoul, Korea). New *Bloc*, *rpb1*, and *tef1* sequences were deposited in GenBank (Table 1). These sequences and others obtained from GenBank were initially aligned with Muscle algorithms (Thompson et al. 1994) and were adjusted manually with MEGA6 software (Tamura et al. 2013).

**Table 1.** List of species used in the molecular analyses.

Species	Country	Strain	*Bloc*	*RPB1*	*tef1*
*B. acridophila*	Colombia	HUA 179219	–	JX003857	JQ958613
	Colombia	HUA 179221	–	JX003853	JQ958615
	Colombia	HUA 179220	–	JX003852	JQ958614
	Colombia	MCA 1181	–	MF416628	–
*B. amorpha*	Australia	ARSEF4149	HQ880735	HQ880876	HQ881006
	USA, Colorado	ARSEF7542	HQ880736	HQ880877	HQ881007
	Chile	B518a	HQ880737	HQ880878	HQ881008
	Peru	ARSEF1969	HQ880738	HQ880879	AY531907
	Brazil	ARSEF2641	HQ880739	HQ880880	AY531917
*B. asiatica*	China	ARSEF4384	HQ880716	HQ880857	AY531935
	China	ARSEF4474	HQ880717	HQ880858	AY531936
	Korea	ARSEF4850	HQ880718	HQ880859	AY531937
*B. australis*	Australia	ARSEF4580	HQ880719	HQ880860	HQ880994
	Australia	ARSEF4622	HQ880721	HQ880862	HQ880996
	Australia	WCN2015	KT961698	HQ880861	HQ880995
*B. bassiana*	Japan	ARSEF1040	HQ880689	HQ880830	AY531881
	Australia	ARSEF300	HQ880690	HQ880831	AY531924
	Italy	ARSEF1564	HQ880692	HQ880833	HQ880974
	Japan	ARSEF7518	HQ880693	HQ880834	HQ880975
	Vietnam	ARSEF751	HQ880694	HQ880831	AY531954
	Brazil	ARSEF1478	HQ880695	HQ880836	AY531890
	Morocco	ARSEF1811	HQ880696	HQ880837	AY531901
*B. brongniartii*	Japan	ARSEF7516	HQ880697	HQ880838	HQ880976
	USA, Oregon	ARSEF10278	HQ880700	HQ880841	HQ880979
	Korea	ARSEF7268	HQ880703	HQ880844	HQ880982
	USA, New York	ARSEF6213	HQ880706	HQ880847	HQ880985
	Japan	ARSEF4363	HQ880707	HQ880848	HQ880986
	Japan	ARSEF4362	HQ880708	HQ880849	HQ880980
	USA, Kentucky	ARSEF2271	HQ880710	HQ880851	HQ880988
	USA, Oregon	ARSEF10277	HQ880711	HQ880852	HQ880989
	France	ARSEF979	HQ880714	HQ880855	HQ880992
*B. caledonica*	Switzerland	ARSEF1567	HQ880747	HQ880888	AY531894
	Scotland	ARSEF2567	HQ880748	HQ880889	AY531915
	Denmark	ARSEF8024	HQ880749	HQ880890	HQ881012
	Brazil	ARSEF2251	HQ880750	HQ880891	AY531912
	USA, Georgia	ARSEF7117	HQ880751	HQ880892	HQ881013
	Australia	ARSEF4302	HQ880752	HQ880893	HQ881014
*B. diapheromeriphila*	Ecuador	QCNE 186272	–	JX003848	JQ958610
	Ecuador	QCNE 186714	–	MF416648	MF416491
	Ecuador	MCA 1557	–	JX003848	JQ958610
*B. hoplocheli*	Reunion	Bt116	KM453967	KM453957	KC339703
	Reunion	Bt121	KM453968	KM453956	KC339704
	Reunion	Bt124	KM453969	KM453955	KC339699
	Reunion	Bt125	KM453970	KM453953	KC339701
	Reunion	Bt128	KM453972	KM453952	KC339705
	Reunion	Bt129	KM453973	KM453951	KC339706
	Madagascar	Bt96	KM453974	KM453950	KC339709
	Reunion	Bt99	KM453975	KM453949	KC339710
*B. kipukae*	USA, Hawaii	ARSEF7032	HQ880734	HQ880875	HQ881005
*B. lii*	China	RCEF5500	JN689373	JN689374	JN689371
*B. malawiensis*	China	GZU12142	MG052638	MG052645	MG052641
	China	GZU12141	MG052639	MG052644	MG052640
	Australia	ARSEF4755	HQ880754	HQ880895	HQ881015
	Australia	BCC17613	HQ880755	HQ880896	HQ881016
	Malawi	ARSEF7760	HQ880756	HQ880897	DQ376246
*B. peruviensis*	Peru	UTRF21	MN094752	MN100113	MN094767
	Peru	UTRF24	MN094753	MN100119	MN094768
	Peru	UTRF25	MN094754	MN100114	MN094769
	Peru	UTRF26	MN094758	MN100120	MN094770
	Peru	UTRF35	MN094755	MN100115	MN094771
	Peru	UTRF37	MN094756	MN100116	MN094772
	Peru	UTRF38	MN094759	MN100121	MN094773
	Peru	UTRF40	MN094760	MN100122	MN094774
	Peru	UTRF42	MN094761	MN100123	MN094775
	Peru	UTRF58	MN094762	MN100124	MN094776
	Peru	UTRP6	MN094763	MN100125	MN094777
	Peru	UTRP7	MN094764	MN100127	MN094778
	Peru	UTRP13	MN094765	MN100126	MN094779
	Peru	UTRP17	MN094766	MN100117	MN094780
	Peru	UTRP19	MN094757	MN100118	MN094781
*B. pseudobassiana*	Portugal	ARSEF3220	HQ880722	HQ880863	AY531928
	USA, Kentucky	ARSEF3405	HQ880723	HQ880864	AY531931
	USA, Wisconsin	ARSEF3216	HQ880725	HQ880866	AY531927
	USA, Maryland	ARSEF3529	HQ880726	HQ880867	HQ880998
	France	ARSEF4933	HQ880726	HQ880870	AY531938
	Canada	ARSEF1855	HQ880727	HQ880868	HQ880999
	Canada	ARSEF2997	HQ880728	HQ880869	HQ881000
	China	ARSEF6229	HQ880730	HQ880871	HQ881001
	Korea	ARSEF7242	HQ880730	HQ880865	HQ880997
*B. scarabaeicola*	Korea	ARSEF5689	–	DQ522380	DQ522335
	Japan	ARSEF1685	HQ880740	HQ880881	AY531899
	Korea	ARSEF5689	HQ880741	HQ880882	AY531939
	Korea	ARSEF7043	HQ880742	HQ880883	AY531948
	Korea	ARSEF7044	HQ880743	HQ880884	AY531949
	Korea	ARSEF7279	HQ880743	HQ880885	HQ881009
	Korea	ARSEF7280	HQ880744	HQ880886	HQ881010
	Korea	ARSEF7281	HQ880746	HQ880887	HQ881011
*B. sinensis*	China	RCEF3903	–	JX524283	HQ270151
*B. staphylinidicola*	Korea	ARSEF5718	–	EF468881	EF468776
*B. varroae*	France	ARSEF8259	HQ880732	HQ880873	HQ881003
	Switzerland	ARSEF2694	HQ880733	HQ880874	HQ881004
	France	ARSEF8257	HQ880733	HQ880872	HQ881002
*B. vermiconia*	Chile	ARSEF2922	HQ880753	HQ880894	AY531920
*Cordyceps cicadae*	Korea	ARSEF7260	HQ880757	HQ880898	HQ881017
*Blackwiella cardinalis*	USA	OSC93610	–	EF469088	EF469059
*Ascopolyporus polychrous*	–	PC546	–	DQ127236	DQ118745

The phylogeny was based on concatenated data combining *Bloc*, *rpb1*, and *tef1* (101 sequences, Table 1). Selection of the best-fitting nucleotide substitution model was conducted using the program PartitionFinder (Lanfear et al. 2012) with three partitions (*Bloc*, *rpb1*, and *tef1*). The best partition strategy and model of sequence evolution were selected based on the Bayesian Information Criterion (BIC). The general time reversible nucleotide substitution model with a gamma distribution and a proportion of invariable sites (GTR + Γ + I) was selected for all partitions. Maximum likelihood (ML) analyses were conducted with the RAxML HPC-AVX program (Stamatakis 2014) implemented in the raxmlGUI 1.3.1 interface (Silvestro and Michalak 2012) using a GTRGAMMAI model with 1000 bootstrap replications. Bayesian inference (BI) was performed with MrBayes v. 3.2.6 software (Ronquist et al. 2012) using Metropolis-coupled MCMC and the GTR + Γ + I model. We conducted two runs each with four chains (three hot and one cold) for 10,000,000 generations, sampling trees every 1,000 generations. We plotted likelihood vs. generation using the Tracer Version v. 1.6 program (Rambaut et al. 2014) to reach a likelihood plateau and set the burn-in value.

### DNA-based species delimitation

Although 26 species have been molecularly confirmed in *Beauveria* (Rehner et al. 2011, Kepler et al. 2017, Chen et al. 2018), only 21 of these species and *Beauveria* sp. from Peru were used in the DNA-based delimitation methods. *Beauveria araneola* W.H. Chen, Y.F. Han, Z.Q. Liang & D.C. Jin, *B. gryllotalpidicola* Luangsa-ard, Ridkaew & Tasanathai, *B. loeiensis* Luangsa-ard, Ridkaew & Tasanathai, *B. medogensis* Imoulan & Y.J. Yao, and *B. rudraprayagi* Y. Agrawal, P. Mual & B.D. Shenoy were not used due to abundant missing data and short sequences for the three markers (e.g. ~731 bp for *rpb1* and ~720 bp for *tef1*).

We explored five different DNA-based delimitation methods using *Bloc*, *rpb1*, and *tef1* data sets to assess species boundaries in *Beauveria*. Although *B. acridophila*, *B. blattidicola* M. Chen, Aime, T.W. Henkel & Spatafora, *B. diapheromeriphila*, *B. locustiphila*, and *B. staphylinidicola* (Kobayasi & Shimizu) B. Shrestha, Kepler & Spatafora lack *Bloc* sequences, these species were used in the analysis to evaluate its status in the new circumscribed *Beauveria*. Two of these DNA-based delimitation methods are based on genetic distance [statistical parsimony network analysis (SPN) (Hart and Sunday 2007) and automatic barcoding gap detection (ABGD) (Puillandre et al. 2012)], two in coalescence [generalized mixed Yule coalescent method (GMYC) (Pons et al. 2006) and Bayesian phylogenetics and phylogeography (BPP) (Rannala and Yang 2003)], and one in genealogical concordance [genealogical concordance phylogenetic species recognition (GCPSR) (Quaedvlieg et al. 2014)]. For the SPN analyses of *Bloc*, *rpb1*, and *tef1*, data sets were generated in TCS 1.21 (Clement et al. 2000) with a maximum connection probability set at 95% statistical confidence. The ABGD method was tested via a web interface (ABGD web, http://wwwabi.snv.jussieu.fr/public/abgd/abgdweb.html). Before analysis, the model criteria were set as follows: variability (P) between 0.001 (Pmin) and 0.1 (Pmax), minimum gap width (X) of 0.1, Kimura-2-parameters and 50 screening steps.

To perform the GMYC delimitation method, an ultrametric tree was constructed in BEAST v.2.0.2 (Drummond et al. 2012), relying on the uncorrelated lognormal relaxed clock, the GTR + Γ + I model, and a coalescent tree prior. Bayesian Markov chain Monte Carlo was run for 50 million generations, and trees and parameters were sampled every 1000 generations. Log files were visualized in Tracer v.1.6 (Rambaut et al. 2014) for assessing the stationary state of parameters on the basis of the value of estimate-effective sample size (ESS). After removing 25% of trees as burn-in, the remaining trees were used to generate a single summarized tree in TreeAnnotator v.2.0.2 (part of the BEAST v.2.0.2 package) as an input file for GMYC analyses. The GMYC analyses with a single threshold model were performed in R (R Development Core Team, http://www.R-project.org) under the ‘splits’ package using the ‘gmyc’ function (R-Forge, http://r-forge.r-project.org/projects/splits/).

To validate the outcomes of single locus species delimitation, a multilocus BPP was applied using the program BP&P v.2.0 (Rannala and Yang 2003, Yang and Rannala 2010, Liu et al. 2015). The three-gene data (*Bloc*, *rpb1*, and *tef1*) were used as input for BPP under the A11 model (A11: species delimitation = 1, species tree = 1). Specimens were *a priori* assigned to species based only on the minimum number of species from the results of the phylogenetic analysis. The guide tree derived from the three-gene ML analysis was used. Five variables (ε1~ε5) were automatically fine-tuned following the instructions of BP&P (Rannala and Yang 2003, Yang and Rannala 2010). The prior distribution of θ and τ could have influenced the posterior probabilities for different models (Yang and Rannala 2010). Analyses were run with three different prior combinations (Leaché and Fujita 2010). Each analysis was run three times to confirm consistency between runs. Two independent MCMC analyses were run for 100,000 generations with the ‘burn-in’ = 20,000.

GCPSR was implemented by identifying independent evolutionary lineages (IELs) and by exhaustive subdivision of strains into phylogenetic species. The criteria used to identify IELs and exhaustive subdivision were the same as those used by Brankovics et al. (2018). These were implemented using Perl scripts developed by Brankovics et al. (2018) and available at GitHub (https://github.com/b-brankovics/GCPSR).

## Results

### Molecular phylogeny

**Figure 2.**
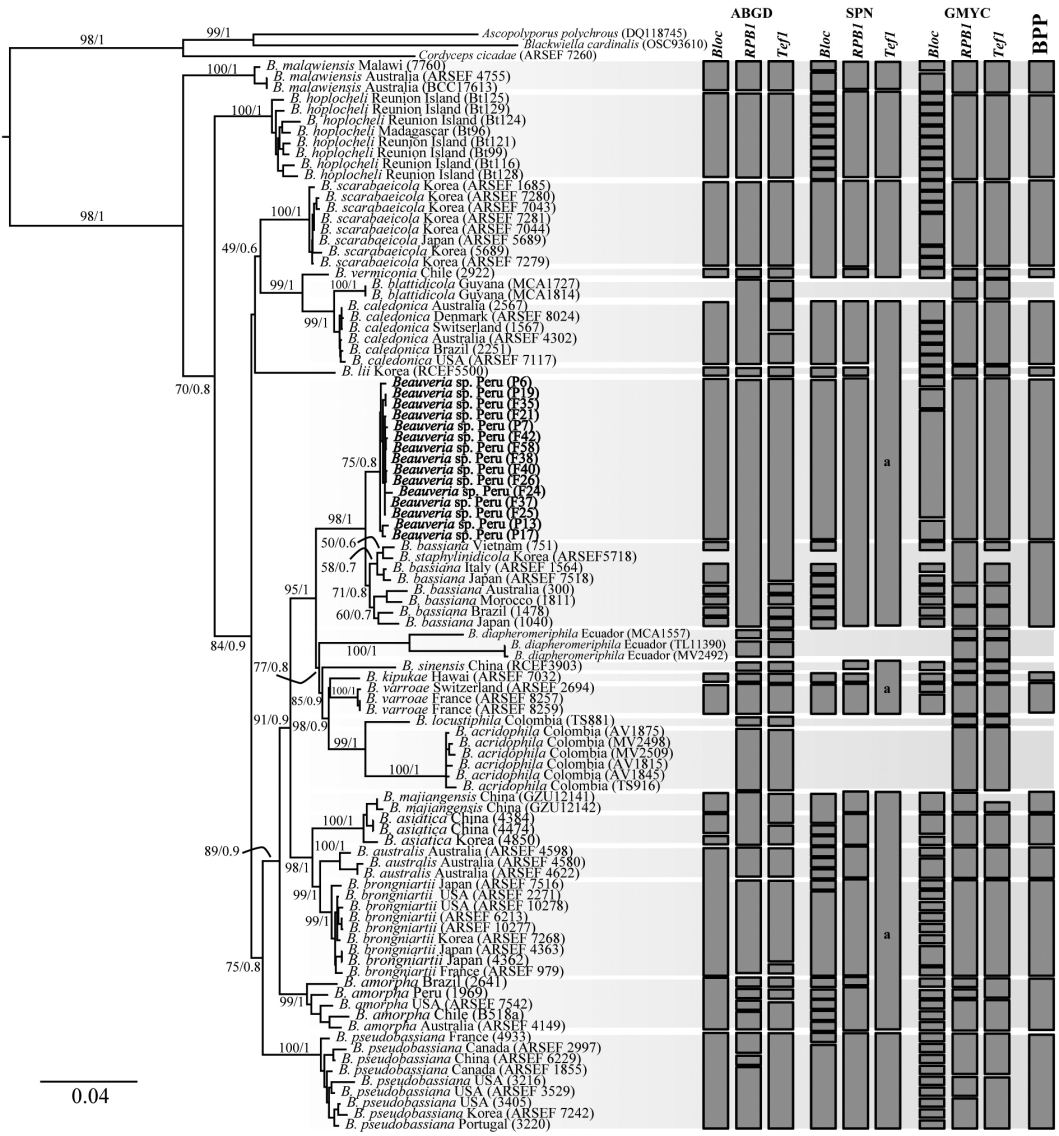
Phylogenetic tree based on maximum likelihood inference of combined *Bloc*, *RPB*1, *Tef*1 data. Value above branches = Maximum likelihood bootstrap values (BS) / Bayesian posterior probabilities. Grey bars represent species delimitation results from ABGD-, SPN-, GMYC- and BPP based algorithmic methods based on *Bloc*, *RPB*1, and *Tef*1 sequences. Scale bar indicates the number of nucleotide substitution per site. a: delimited as the same species. *B. araneola*, *B. gryllotalpidicola*, *B. loeiensis*, *B. medogensis*, and *B. rudraprayagi* were not delimited by any DNA-based algorithm due to abundant missing data in their sequences.

In the phylogeny of *Beauveria* species, the analyzed data matrix included 1592 base pairs (bp) for *Bloc*, 2890 bp *rpb1*, and 1181 bp for *tef1* of 101 individuals. Phylogenetic trees obtained from ML and BI analyses confirmed the robustly supported monophyly of the genus *Beauveria* (Fig. 2). The tree topologies for the individual genes (*tef1*, *Bloc*, and *rpb1*) did not show congruence (Suppl. material 1: Figs S1–S3). These trees showed topological differences, especially in the clades composed of *B. asiatica* / *B. majiangensis* and by *B. bassiana* / *B. staphylinidicola* / *Beauveria* sp. from Peru. Although the individual gene trees did not show congruence with the combined data, the latter resolved these clades, suggesting conspecificity in the first clade and sister relationship in the second. Moreover, the multilocus phylogeny showed well-supported clades in both the ML and BI analyses except in *B. lii*, *B. majiangensis*, and *B. staphylinidicola*. The genetic divergence comparisons showed that the minimum threshold (p-distance) to distinguish genetic species in *Beauveria* was 1.3%, 0.4%, and 0.2% for *Bloc*, *rpb1*, and *tef1*, respectively, as occurred between *B. australis* and *B. asiatica*.(Table 2).

**Table 2.** Genetic distance (p-distances) in percentage for species of *Beauveria* for three markers.

**Taxa**	**Markers**		
	***Bloc***	***RPB*1**	***tef1***
*B. australis* – *B. asiatica*	1.3	0.4	0.2
*B. bassiana* – *B. staphylinidicola*	3.1	0.5	0.2
*B. bassiana* – *B. peruviensis*	3.5–4.1	0.3–0.5	0.2–0.4
*B. peruviensis* – *B. staphylinidicola*	4.1–4.7	0.7–1.1	0.2

### Species delimitation

The species-delimitation methods based on genetic distance (ABGD, SPN), coalescence (GMYC, BPP), and genealogical concordance (GCPSR) showed incongruent results for the three genes (Fig. 2, Table 3). Among these methods, the highest number of species was delimited in the GMYC analysis for the *Bloc* gene, whereas conservative results were observed in BPP. The species delimitations by SPN and GCPSR have inadequate and contradictory results. The genetic distance method based on the barcode gap (ABGD) found similar species numbers for *Bloc*, *rpb1*, and *tef1*, differing only in the species recognized in the clades *B. asiatica* / *B. majiangensis* and *B. bassiana* / *B. staphylinidicola* / *Beauveria* sp. from Peru. In the former clade, there were 3, 1 and 2 species for *Bloc*, *rpb1*, and *tef1*; whereas in the latter clade, there were 7, 1, and 5 species for *Bloc*, *rpb1*, and *tef1*. The GMYC identified relatively conserved results in *RPB*1 (28) and *tef1* (26) and plenty of species in the *Bloc* data set (63). This high number of species for the *Bloc* data set is a consequence of the splitting of the main clades into different species but lacking significance (Suppl. material 1: Table S1, Fig. S4). Regarding the multi-locus coalescent species validation (BPP), the highest posterior probabilities for *Bloc*, *rpb1*, and *tef1* were found by recognizing 16 species based on the results from the phylogenetic analysis and single species delimitation methods (Suppl. material 1: Table S2). Conversely, the BPP analyses with the maximum number of species (39 and 62) were not used based on the inadequate results from SPN and GCPSR. Although there were incongruent results among different methods, the conservative results from species delimitation methods (ABGD and GMYC) and phylogenetic analysis suggest that *Beauveria* is composed of 20–28 and 26 species, respectively. These results also suggest that the clade composed of *B. asiatica* / *B. majiangensis*, *B. diapheromeriphila*, and *B. bassiana* / *B. staphylinidicola* / *Beauveria* sp. from Peru were genetically composed of more than one species. Our analysis also revealed that *Beauveria* sp. from Peru was supported as a distinct species by ABGD (*Bloc* gene), GMYC, BPP, and phylogeny. Thereby, the description of *Beauveria* sp. as a new species is proposed.

**Table 3.** Species number in *Beauveria* identified under DNA-based species-delimitations methods and phylogeny.

Taxa	Genetic distance						Coalescence				Genealogical concordance	Phylogeny
	ABGD			SPN			GMYC			BPP	GCPSR	
	*Bloc*	*RPB*1	*Tef1*	*Bloc*	*RPB*1	*Tef1*	*Bloc*	*RPB*1	*Tef1*			
*B. acridophila*	–	1	1	–	x	x	–	1	1	–	1	1
*B. amorpha*	1	4	3	5	2	x	5	3	2	1		1
*B. asiatica*	2	x	1	2	1	x	2	1	1	1		1
*B. australis*	x	1	1	3	1	x	2	1	1	1		1
*B. bassiana*	6	x	5	6	x	x	6	3	3	1		1
*B. blattidicola*	–	x	1	–	x	x	–	1	1	–		1
*B. brongniartii*	x	1	2	2	1	x	8	1	1	1		1
*B. caledonica*	1	1	2	1	1	x	6	1	1	1		1
*B. diapheromeriphila*	–	2	2	–	x	x	–	2	2	–		1
*B. hoplocheli*	1	1	1	8	1	1	8	1	1	1		1
*B. kipukae*	1	1	1	1	1	x	1	1	1	1		1
*B. lii*	1	1	1	1	1	x	1	1	1	1		1
*B. locustiphila*	–	1	1	–	x	x	–	1	1	–		1
*B. majiangensis*	1	x	x	x	1	x	1	1	1	1		1
*B. malawiensis*	1	1	1	2	1	1	2	1	1	1		1
*B. pseudobassiana*	1	3	1	2	1	1	9	3	2	1		1
*B. scarabaeicola*	1	1	1	x	1	x	6	1	1	1		1
*B. sinensis*	–	1	1	–	1	x	1	1	1	–		1
*B. staphylinidicola*	–	x	x	–	x	x	–	x	x	–		1
*B. varroae*	1	1	1	1	1	x	2	1	1	1		1
*B. vermiconia*	1	1	1	x	1	x	1	1	1	1		1
*B. peruviensis*	1	x	x	1	x	x	2	1	1	1		1
Total	20	22	28	35	16	3	63	28***	26***	16*	1	22

x = non recognized as species, - = not evaluated, * = posterior probabilities higher or equal than 0.53, *** =highly significant

### Morphological observations

#### Beauveria peruviensis

Taxon classificationFungiHypocreales Cordycipitaceae

D.E.Bustamante, M.S.Calderon, M.Oliva, S.Leiva sp. nov.

0499423D9E175E25A78F332AB62C432D

MycoBank No: 829032

Fig. 3

##### Diagnosis.

Species very similar morphologically to *Beauveria bassiana*, but differing in the sister phylogenetic relationship with this species (Fig. 2). The sequence divergence between *B. peruviensis* and *B. bassiana* is 3.5–4.1% for *Bloc*, 0.3–0.5% for *rpb1*, and 0.2–0.4% for *tef1*. *B. peruviensis* is occurring in coffee plantations located in the middle altitudes of the Amazon region of Peru.

**Figure 3.**
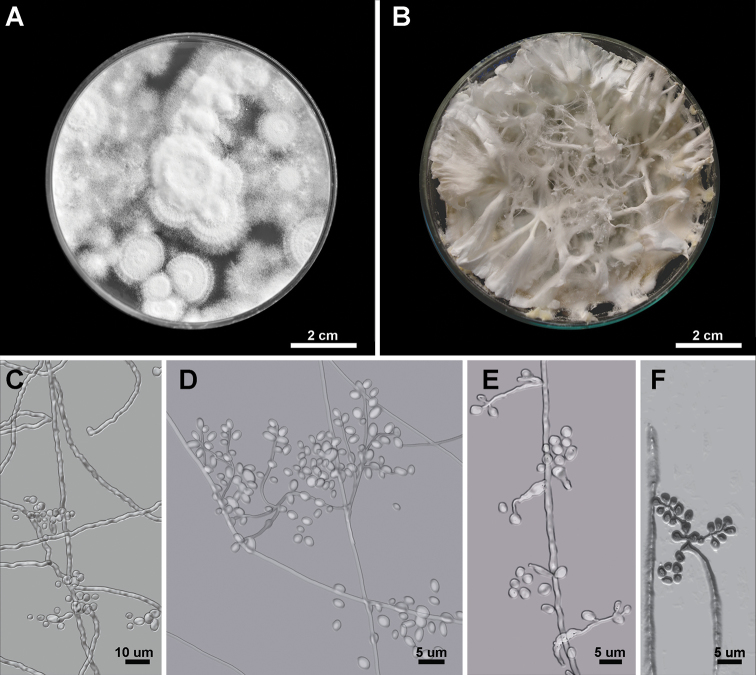
Morphology of *Beauveria amazonensis*. **A, B** Colony growth on PDA showing the habit **C–F** conidiogenous cells and conidia.

##### Type.

PERU. Amazonas: Prov. Rodríguez de Mendoza, Dist. Huambo, latitude -6.469, longitude -77.376, elev. 1642 m, entomopathogenic, 08 Nov. 2017, G. Ángulo, UTRP19 (holotype: UFV5609; isotype: ARSEF14196).

##### Description.

Colony growth on PDA, 15–38 mm diam. after 15 d at 25 C, 1.4–1.9 daily rate of radial growth, velutinous and closely appressed to agar surface, up to 3.5 mm thick, white, changing to yellowish white in older sections of the colony. Conidia aggregated as ca. 0.1 mm spherical clusters and white in mass. Colony reverse colorless or yellowish white to grayish white. Odor indistinct. Vegetative hyphae septate, branched, hyaline, smooth-walled, 1–1.5 μm wide. Conidiogenous cells, phialidic, solitary or occurring in dense lateral clusters, base subsphaerical, 3–6 μm wide, sympodially branched neck tapering into a long slender denticulate rachis, geniculate or irregularly bent, 2.0–3.5 × 1.5–2.5 μm. Conidia, 2–3 × 1–3 μm, Q = 1.0–1.8 (L^m^ = 2.5 μm, W^m^ = 2.2 μm, Q^m^ = 1.6), mainly globose, slightly ellipsoid, oblong or cylindrical, hyaline, aseptate, walls smooth and thin. Mycelium on the host is granular-pulverulent, sometimes funiculose or rarely producing synnemata, white, rarely yellowish. Hyphae of the aerial mycelium bearing a conidial apparatus as described above. Basal parts of the conidiogenous cells globose, subglobose or somewhat flask-shaped.

##### Distribution.

This species is widely spread on coffee plantations in the middle altitudes of the Amazon region in northeastern Peru.

##### Ecology.

*B. peruviensis* was isolated from coffee borers (*Hypothenemus hampei*) obtained from coffee grains. Only the asexual stage was found.

##### Etymology.

The specific epithet ‘*peruviensis*’ is derived from the country where the samples were collected.

##### Additional specimens examined.

PERU. Amazonas: Prov. Rodríguez de Mendoza, Dist. Chirimoto, Achamal, -6.535, -77.408, 1351 m alt., 26 Jul. 2017, G. Angulo UTRF21 (UTR) ; -6.534, -77.409, 1345 m alt., 26 Jul. 2017, G. Angulo UTRF22 (UTR) ; -6.544, -77.404, 1435 m alt., 26 Jul. 2017, G. Angulo UTRF23 (UTR); -6.539, -77.401, 1374 m alt., 26 Jul. 2017, G. Angulo UTRF24 (UTR); -6.539, -77.407, 1386 m alt., 26 Jul. 2017, G. Angulo UTRF25 (UTR); -6.543, -77.405, 1428 m alt., 26 Jul. 2017, G. Angulo UTRF26 (UTR); Paraiso, -6.569, -77.383, 1218 m alt., 26 Jul. 2017, G. Angulo UTRF37 (UTR); -6.568, -77.382, 1197 m alt., 26 Jul. 2017, G. Angulo UTRF38 (UTR); -6.567, -77.389, 1387 m alt., 26 Jul. 2017, G. Angulo UTRF39 (UTR); -6.571, -77.385, 1250 m alt., 26 Jul. 2017, G. Angulo UTRF40 (UTR); -6.579, -77.403, 1427 m alt., 10 Aug. 2017, G. Angulo UTRP12 (UTR); -6.58, -77.403, 1444 m alt., 10 Aug. 2017, G. Angulo UTRP13 (UTR); -6.579, -77.404, 1439 m alt., 10 Aug. 2017, G. Angulo UTRP14 (UTR); Trancapata, -6.546, -77.389, 1255 m alt., 26 Jul. 2017, G. Angulo UTRF31 (UTR); -6.564, -77.384, 1161 m alt., 26 Jul. 2017, G. Angulo UTRF34 (UTR); Virgen del Carmen, -6.586, -77.379, 1313 m alt., 26 Jul. 2017, G. Angulo UTRF42 (UTR); -6.586, -77.378, 1271 m alt., 26 Jul. 2017, G. Angulo UTRF43 (UTR); -6.586, -77.377, 1256 m alt., 26 Jul. 2017, G. Angulo UTRF44 (UTR); -6.581, -77.377, 1138 m alt., 26 Jul. 2017, G. Angulo UTRF46 (UTR); Zarumilla, -6.568, -77.376, 1118 m alt., 26 Jul. 2017, G. Angulo UTRF35 (UTR); -6.58, -77.403, 1461 m alt., 10 Aug. 2017, G. Angulo UTRP15 (UTR); -6.58, -77.403, 1149 m alt., 10 Aug. 2017, G. Angulo UTRP16 (UTR); -6.559, -77.385, 1160 m alt., 10 Aug. 2017, G. Angulo UTRP17 (UTR); -6.558, -77.385, 1160 m alt., 10 Aug. 2017, G. Angulo UTRP18 (UTR); Huambo, Chontapamapa, -6.419, -77.557, 1637 m alt., 27 Jul. 2017, G. Angulo UTRF66 (UTR); Dos Cruces, -6.579, -77.378, 1624 m alt., 27 Jul. 2017, G. Angulo UTRF53 (UTR); -6.424, -77.548, 1668 m alt., 27 Jul. 2017, G. Angulo UTRF58 (UTR); -6.425, -77.55, 1642 m alt., 11 Aug. 2017, G. Angulo UTRP19 (UTR); -6.425, -77.55, 1629 m alt., 11 Aug. 2017, G. Angulo UTRP20 (UTR); -6.424, -77.549, 1661 m alt., 11 Aug. 2017, G. Angulo UTRP21 (UTR); -6.425, -77.548, 1671 m alt., 11 Aug. 2017, G. Angulo UTRP22 (UTR); -6.424, -77.548, 1681 m alt., 11 Aug. 2017, G. Angulo UTRP23 (UTR); -6.423, -77.548, 1682 m alt., 11 Aug. 2017, G. Angulo UTRP24 (UTR); -6.422, -77.548, 1671 m alt., 11 Aug. 2017, G. Angulo UTRP25 (UTR); Escobar, -6.42, -77.549, 1666 m alt., 27 Jul. 2017, G. Angulo UTRF59 (UTR); -6.42, -77.549, 1674 m alt., 27 Jul. 2017, G. Angulo UTRF60 (UTR); Omia, El Tingo, -6.469, -77.376, 1431 m alt., 25 Jul. 2017, G. Angulo UTRF19 (UTR); -6.475, -77.381, 1349 m alt., 25 Jul. 2017, G. Angulo UTRF20 (UTR); La Primavera, -6.634, -77.231, 1283 m alt., 25 Jul. 2017, G. Angulo UTRF5 (UTR); -6.64, -77.224, 1362 m alt., 25 Jul. 2017, G. Angulo UTRF7 (UTR); -6.632, -77.222, 1205 m alt., 3 Aug. 2017, G. Angulo UTRP4 (UTR); -6.632, -77.222, 1209 m alt., 3 Aug. 2017, G. Angulo UTRP5 (UTR); -6.638, -77.225, 1280 m alt., 3 Aug. 2017, G. Angulo UTRP6 (UTR); -6.637, -77.225, 1275 m alt., 3 Aug. 2017, G. Angulo UTRP7 (UTR); -6.636, -77.227, 1255 m alt., 25 Jul. 2017, G. Angulo UTRP8 (UTR); -6.632, -77.225, 1238 m alt., 4 Aug. 2017, G. Angulo UTRP9 (UTR); Libano, -6.623, -77.235, 1174 m alt., 24 Jul. 2017, G. Angulo UTRF2 (UTR); -6.611, -77.237, 1330 m alt., 24 Jul. 2017, G. Angulo UTRF3 (UTR); -6.625, -77.242, 1235 m alt., 24 Jul. 2017, G. Angulo UTRF4 (UTR); -6.612, -77.237, 1307 m alt., 3 Aug. 2017, G. Angulo UTRP1 (UTR); -6.618, -77.234, 1242 m alt., 3 Aug. 2017, G. Angulo UTRP2 (UTR); -6.626, -77.247, 1284 m alt., 3 Aug. 2017, G. Angulo UTRP3 (UTR); -6.62, -77.235, 1226 m alt., 3 Aug. 2017, G. Angulo UTRP10 (UTR); -6.618, -77.237, 1236 m alt., 4 Aug. 2017, G. Angulo UTRP11 (UTR).

##### Notes.

*Beauveria peruviensis* is practically indistinguishable in morphology to other *Beauveria* species. The shape and size of the conidia and the colony color of *B. peruviensis* among other morphological features have been observed in *B. bassiana*, *B. kipukae*, *B. pseudobassiana*, and *B. varroae* (Rehner et al. 2011). The lack of diagnostic morphological features to distinguish *Beauveria peruviensis* was overcome by delimiting this species with DNA-based methodologies.

## Discussion

Accurate species identification within the entomopathogenic fungi *Beauveria* is crucial for disease control and prevention (Liu et al. 2016). This genus has recently been circumscribed, and its taxonomy has been updated with new combinations and the description of new species based mainly on multi-locus phylogenies in the absence of diagnostic features that delimit species (Sanjuan et al. 2014, Shrestha et al. 2014, Kepler et al. 2017, Chen et al. 2017, 2018). In addition to phylogenies, other methodologies and data sets to delimit species are recommended to establish well-supported boundaries among species (Carstens et al. 2013) because most researchers simply recognize distinct clades in either single- or multi-locus trees as species (Stewart et al. 2014). In this regard, our study used phylogeny and five DNA-based methods to delimit species in *Beauveria* using three molecular makers. Although incongruence among some of these methods was observed in our analyses, a genetic distance (ABGD), a coalescence method (BPP), and the multilocus phylogeny strongly supported 20–28 different species, including the new species *B. peruviensis* from Peru.

The use of multi-locus sequence data is essential to establish robust species boundaries (Lumbsch and Levitt 2011), and our results for *Beauveria* showed well-supported clades, although it resulted in incongruence to the single locus phylogenies (Suppl. material 1: Figs S1–S3). This conflict can be a result of incomplete lineage sorting, horizontal gene transfer, gene duplication and loss, hybridization, or recombination (Degnan and Rosenberg 2009). This study cannot determine which of these scenarios are occurring in *Beauveria*; nevertheless, it serves as a baseline for investigating causes of gene tree discordance that can be identified by further analyses at the genomic level (Patterson et al. 2006, Liu et al. 2016). According to our multilocus phylogeny, 22 of the 26 molecularly confirmed species in *Beauveria* were recognized. Previous studies have delimited *B. araneola*, *B. gryllotalpidicola*, *B. loeiensis*, *B. medogensis*, and *B. rudraprayagi* as valid species on the basis of their phylogenies (Agrawal et al. 2014, Imoulan et al. 2016, Chen et al. 2017); however, our study did not include these sequences because they have abundant missing data, and thus, their status was not evaluated. These species would require further revision to be recognized as supported lineage within the genus *Beauveria*.

Regarding the genetic distance methods, the ABGD showed similar results when delimiting *Beauveria* species to those from the multilocus phylogeny. The additional putative species in ABGD is mainly due to the split of *B. bassiana*. This confirms that *B. bassiana* encompasses cryptic lineages as proposed initially by Rehner et al. (2011). Therefore, the original *B. bassiana* should be the clade that includes the specimen from the type locality, namely, Italy (Vuillemin 1912). Additionally, these results delimited *B. majiangensis* and *B. asiatica* as different lineages, although the multilocus analysis showed low support. *B. majiangensis* needs further analysis with additional and longer sequences to confirm its status because one or only a few individuals often fail to represent the species as a whole (Davis and Nixon 1992, Walsh 2000). On the other hand, the SPN method showed conflicting results among the *Bloc*, *RPB*1, and *tef1* loci, leading to incorrect inferences. The number of species inferred by SPN greatly matched the number of Linnaean species in mitochondrial markers (e.g., COI) (Hart and Sunday 2007). Therefore, our nuclear markers due to indels might generate many reticulations that allow inadequate species delimitation in our data (Paradis 2018).

In the coalescence methods, although 6 species were not included in the BPP analysis due to the lack of their *Bloc* sequences, this method supports the conservative results obtained from the multilocus phylogeny. BPP supported the status of 16 species (posterior probabilities higher than 0.52), which are not high supportive, but these probabilities are not supportive at all when splitting or merging species in the BPP analysis (Suppl. material 1: Table S2). Zhang et al. (2011) found that the correct species model was inferred with a high posterior probability with only one or two loci when 5 or 10 sequences were sampled from each population or with 50 loci when only one sequence was sampled, and they also demonstrated that the migration rate might affect these results. This suggests that further analysis might need to increase the number of sequences per locus among different populations of species of *Beauveria* and assess their migration rate to obtain supportive delimitations. Moreover, the highly significant results obtained from the GMYC method for the *tef1* and *rpb1* loci partially support the ABGD and multilocus analyses. The additional number of putative species in the GMYC analyses, as occurred with the ABGD, is due to the presence of more than one lineage in *B. amorpha*, *B. bassiana*, *B. diapheromeriphila*, and *B. pseudobassiana* confirming cryptic diversity (Rehner et al. 2011). The performance in empirical studies of the ABGD and GMYC tends to under- and oversplit species, respectively (Luo et al. 2018). However, our results suggest that GMYC and ABGD are appropriate for determining cryptic diversity in *Beauveria* by splitting well-supported clades from the multi-locus phylogeny.

Regarding *B. peruviensis*, ABGD (*Bloc*), SPN (*Bloc*), GMYC, BPP, and the phylogenetic analyses support this species as a different lineage from *B. bassiana* and *B. staphylinidicola*. Additionally, the genetic divergence between *B. peruviensis* and these species is higher than the minimum threshold observed in species of *Beauveria* (Table 2). In our study, *Beauveria peruviensis* showed morphological indistinctiveness to other *Beauveria* species that produce globose/subglobose/ellipsoid conidia. Additionally, *B. peruviensis* conidia is also similar in size to other *Beauveria*, especially *B. bassiana*. Previously, Rehner et al. (2011) noted that *B. bassiana* is hardly distinguishable from other species of *Beauveria*. The lack of diagnostic morphological features to delimit species in *Beauveria* was overcome by the application of molecular methods in fungal taxonomy. The segregation of *B. peruviensis* from *B. bassiana* and *B. staphylinidicola* confirmed that phylogenetic diversity and DNA-species delimitation methods discover taxa within morphologically defined species (Goldstein et al. 2000, Liu et al. 2016). Ecologically, the segregation of *B. peruviensis* from *B. bassiana* and *B. staphylinidicola* is supported by the specificity of *B. peruviensis* to the coffee borer from Amazon and the well-supported lineage in the phylogenetic analysis that might indicate the presence of a barrier in gene flow in nature (Van Valen 1976, Liu et al. 2016).

Recently, polyphasic approaches have been used to reflect the natural classification of species within many important fungal genera (Aveskamp et al. 2010, Milic et al. 2012, Liu et al. 2016). These approaches frequently incorporate morphological and phylogenetic analyses and metabolomics, but few of them use genetic distance and coalescent methods (Liu et al. 2016). The use of polyphasic analysis, including DNA-based delimitation methods, allowed the establishment of boundaries among species of morphologically conserved genera such as *Beauveria* and thus provided support for the description of new taxa (e.g., *B. peruviensis*) or validated the taxonomic uncertain of others (e.g., *B. majiangensis*). Although more recent methods avoid arbitrary cut-offs (Knowles and Carstens 2007), our results demonstrate that the congruence among this method and other methods used in a polyphasic approach (e.g., genetic distance, coalescence methods) are more likely to prove reliably supported species boundaries (Carstens et al. 2013). Among the methods applied in this study, ABGD, GMYC, BPP, and multilocus phylogeny are crucial when establishing species boundaries in *Beauveria*.

## Supplementary Material

XML Treatment for Beauveria peruviensis

## Supplementary materials

Supplementary material 1 Tables S1, S2, Figures S1–S4 Data type: molecular data Explanation note: **Table S1.** Results of the Generalized Mixed Yule-Coalescent (GMYC) analyses under the single threshold model. **Table S2.** Highest posterior probabilities of the three-gene Bayesian species delimitation analysis (BPP) by jointing species delimitation and species tree inference. **Figure S1.** Phylogenetic tree based on maximum likelihood inference of combined *Bloc* data. **Figure S2.** Phylogenetic tree based on maximum likelihood inference of combined *RPB*1 data. **Figure S3.** Phylogenetic tree based on maximum likelihood inference of combined *Tef*1 data. **Figure S4.** Bayesian inference ultrametric gene tree. https://binary.pensoft.net/file/338791This dataset is made available under the Open Database License (http://opendatacommons.org/licenses/odbl/1.0/). The Open Database License (ODbL) is a license agreement intended to allow users to freely share, modify, and use this Dataset while maintaining this same freedom for others, provided that the original source and author(s) are credited.Danilo E. Bustamante, Manuel Oliva, Santos Leiva, Jani E. Mendoza, Leidy Bobadilla, Geysen Angulo, Martha S. Calderon
