# Supplementary material for: Phylogeny and species delimitations in the entomopathogenic genus Beauveria (Hypocreales, Ascomycota), including the description of B. peruviensis sp. nov
Source: MycoKeys. 2019 Sep 9;58:47–68. doi: 10.3897/mycokeys.58.35764 (PMC6746742; doi:10.3897/mycokeys.58.35764)
Supplement: Supplementary material 1 [file mycokeys-58-047-s001.docx]

Supplemental Material

**Table S1.** Results of the Generalized Mixed Yule-Coalescent (GMYC) analyses under the single threshold model.

| **Gene** | ***N*_CLUSTER_ (CI)** | ***N*_GMYC_ (CI)** | ***L*_NULL_** | ***L*_GMYC_** | **Λ** | ***P*** | **Threshold** |
| --- | --- | --- | --- | --- | --- | --- | --- |
| *Bloc* | 11 (2-16) | 63 (2-65) | 477.524 | 479.895 | 4.7423 | 0.58929e-07n.s. | -0.0008098 |
| *RPB*1 | 20 (18-21) | 28 (26-31) | 795.954 | 803.604 | 15.3006 | 0.0004759011*** | -0.0027721 |
| *tef*1 | 18 (18-19) | 26 (25-27) | 785.479 | 798.443 | 25.9281 | 2.343082e-06*** | -0.0034657 |

Abbreviation: *N*_CLUSTER_, number of GMYC lineages with more than one specimens; *N*_GMYC_, number of GMYC lineages; CI, confidence interval; *L*_NULL_, likelihood of the null model; *L*_GMYC_, likelihood of the GMYC model; Λ, likelihood ratio; *P*, *P* values of the likelihood ratio test; Threshold, the threshold line between speciation and coalescent process.

**Table S2.** Highest posterior probabilities of the three-gene Bayesian species delimitation analysis (BPP) by jointing species delimitation and species tree inference (A11: species delimitation = 1, species tree = 1)

| **Delimited species** | **Run 1** | **Run 2** | **Run 3** | **Average** |
| --- | --- | --- | --- | --- |
| *B. amorpha* | 0.486 | 0.601 | 0.534 | 0.540 |
| *B. asiatica* | 0.532 | 0.565 | 0.510 | 0.536 |
| *B. australis* | 0.491 | 0.556 | 0.534 | 0.527 |
| *B. bassiana* | 0.506 | 0.550 | 0.566 | 0.540 |
| *B. brongniartii* | 0.538 | 0.564 | 0.534 | 0.545 |
| *B. caledonica* | 0.501 | 0.573 | 0.555 | 0.543 |
| *B. hoplocheli* | 0.531 | 0.564 | 0.516 | 0.537 |
| *B. kipukae* | 0.502 | 0.588 | 0.510 | 0.533 |
| *B. lii* | 0.508 | 0.559 | 0.513 | 0.527 |
| *B. majiangensis* | 0.537 | 0.585 | 0.528 | 0.550 |
| *B. malawiensis* | 0.505 | 0.574 | 0.571 | 0.550 |
| *B. pseudobassiana* | 0.547 | 0.530 | 0.529 | 0.535 |
| *B. sungii* | 0.508 | 0.551 | 0.544 | 0.534 |
| *B. varroae* | 0.533 | 0.592 | 0.569 | 0.564 |
| *B. vermiconia* | 0.533 | 0.548 | 0.524 | 0.535 |
| *Beauveria sp.* | 0.499 | 0.559 | 0.531 | 0.529 |
| Splitting a species | 0.029 | 0.033 | 0.026 | 0.029 |
| More than 2 species cluster | 0.031 | 0.025 | 0.030 | 0.029 |

**
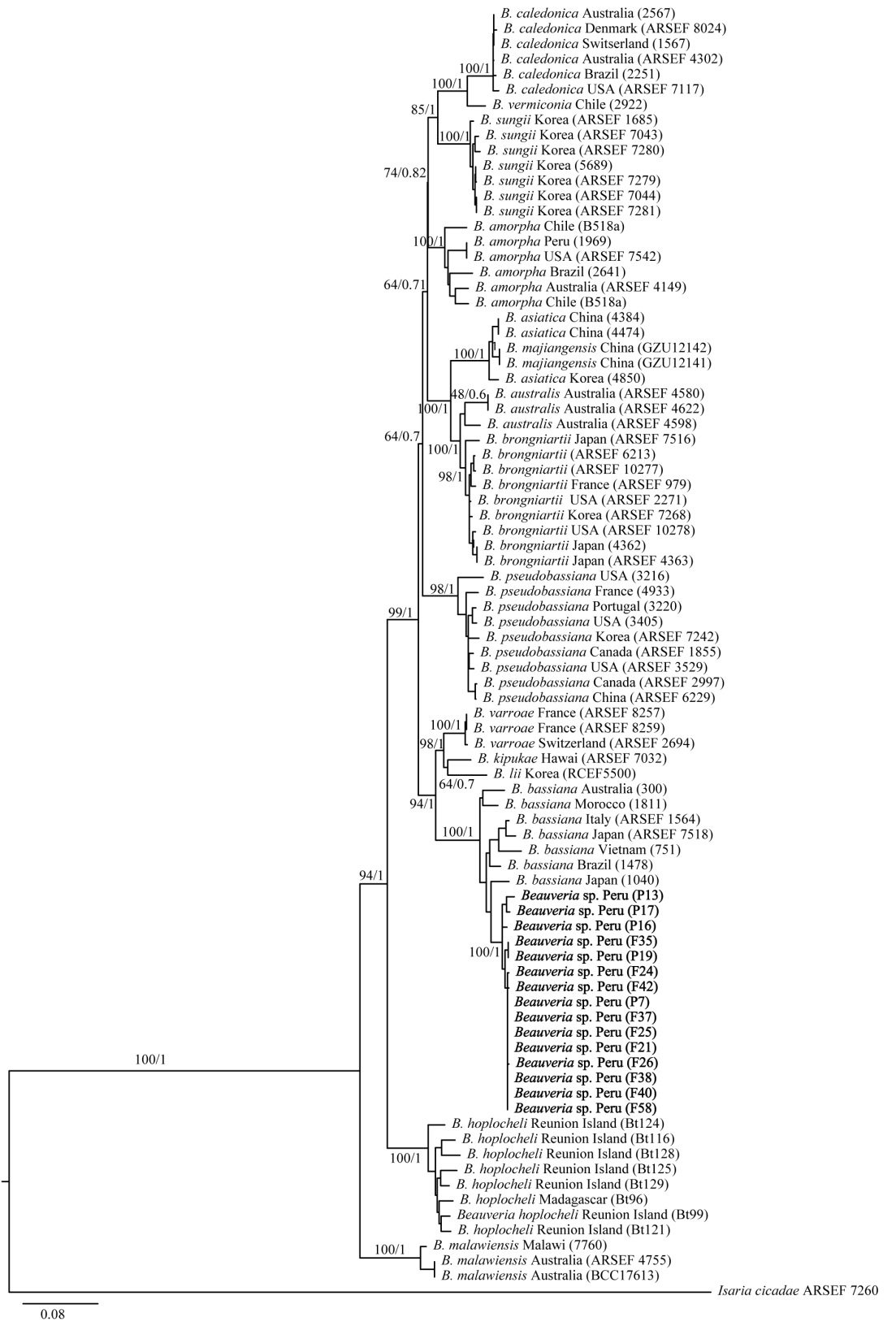
**

**Fig. S1.** Phylogenetic tree based on maximum likelihood inference of combined *Bloc* data. Value above branches = Maximum likelihood bootstrap values (BS) / Bayesian posterior probabilities. Scale bar indicates the number of nucleotide substitution per site.

**
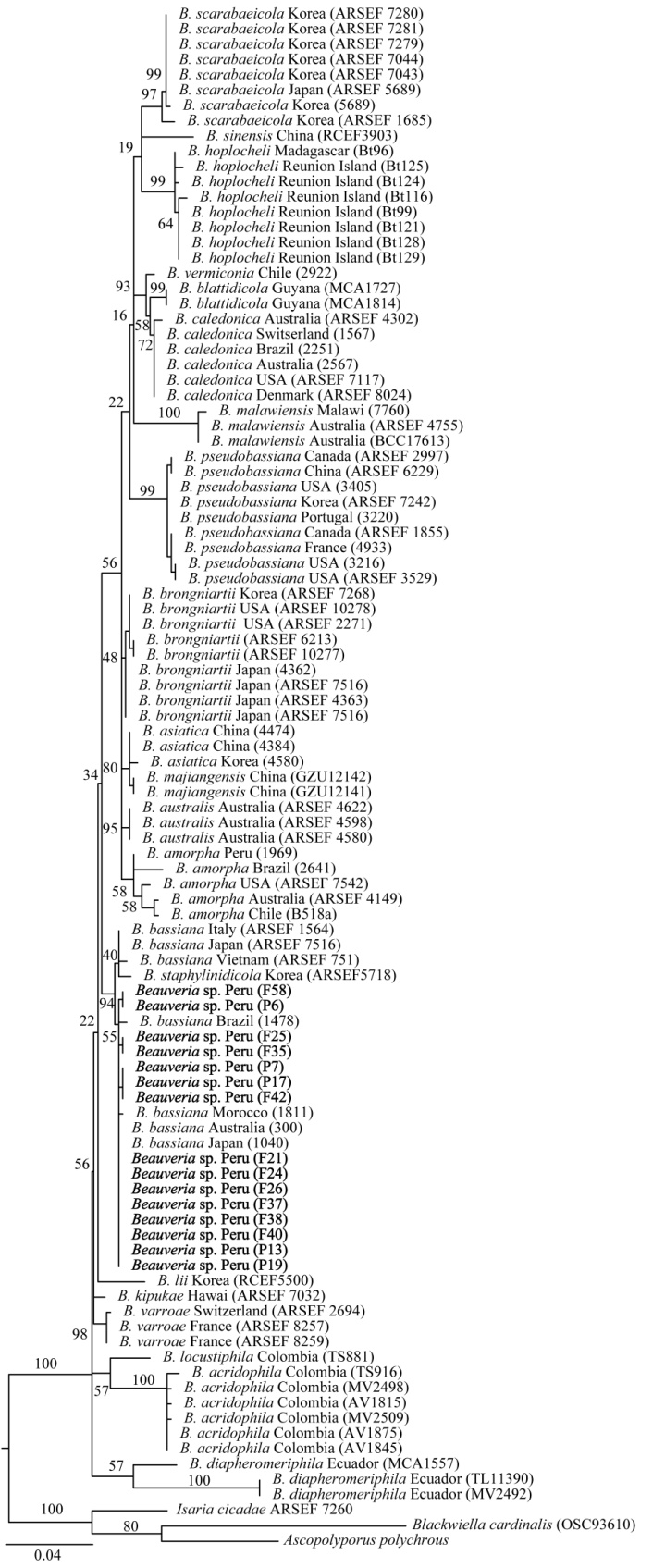
**

**Fig. S2.** Phylogenetic tree based on maximum likelihood inference of combined *RPB*1 data. Value above branches = Maximum likelihood bootstrap values (BS). Scale bar indicates the number of nucleotide substitution per site.

**
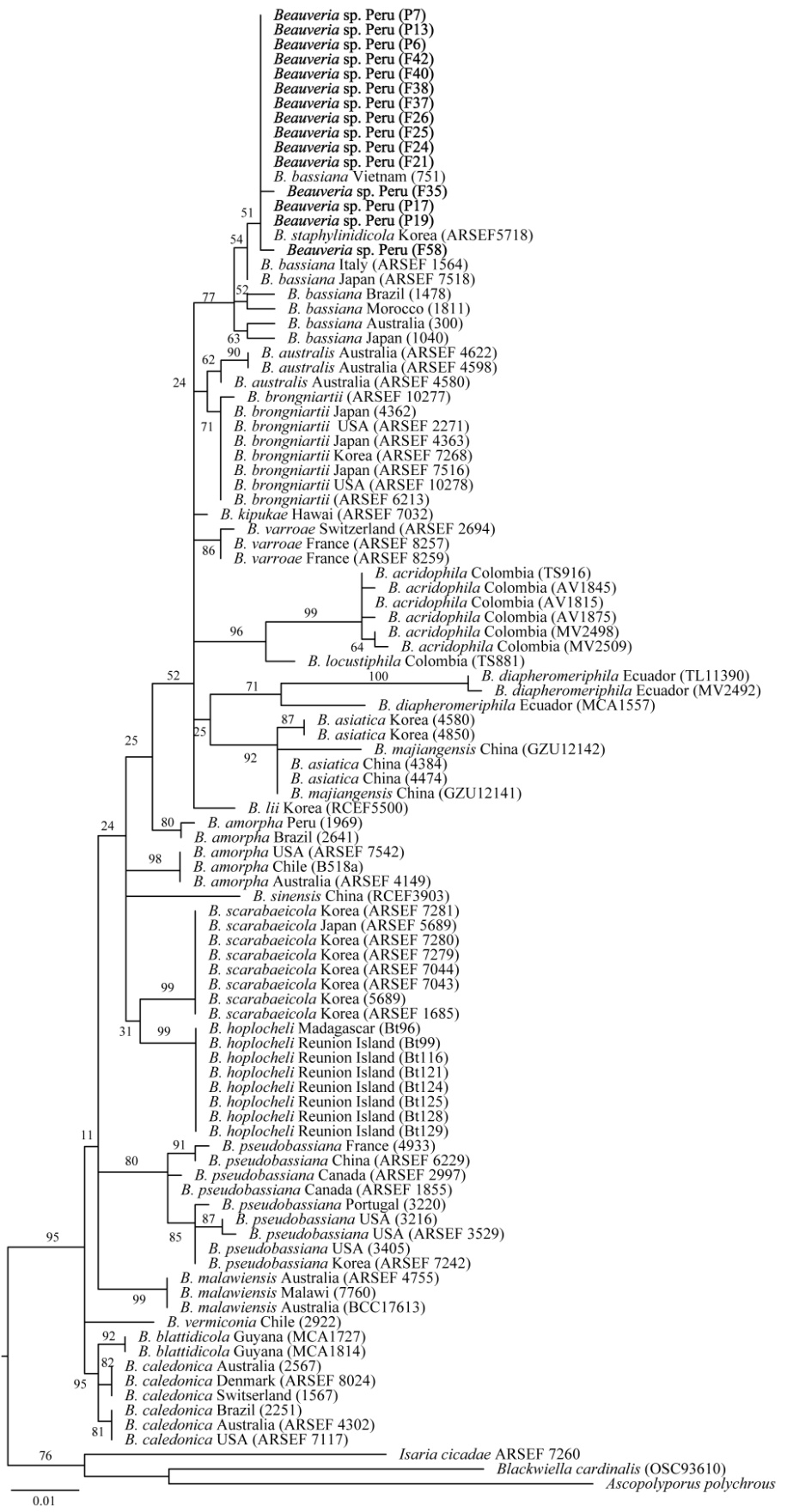
**

**Fig. S3.** Phylogenetic tree based on maximum likelihood inference of combined *Tef*1 data. Value above branches = Maximum likelihood bootstrap values (BS). Scale bar indicates the number of nucleotide substitution per site.

**
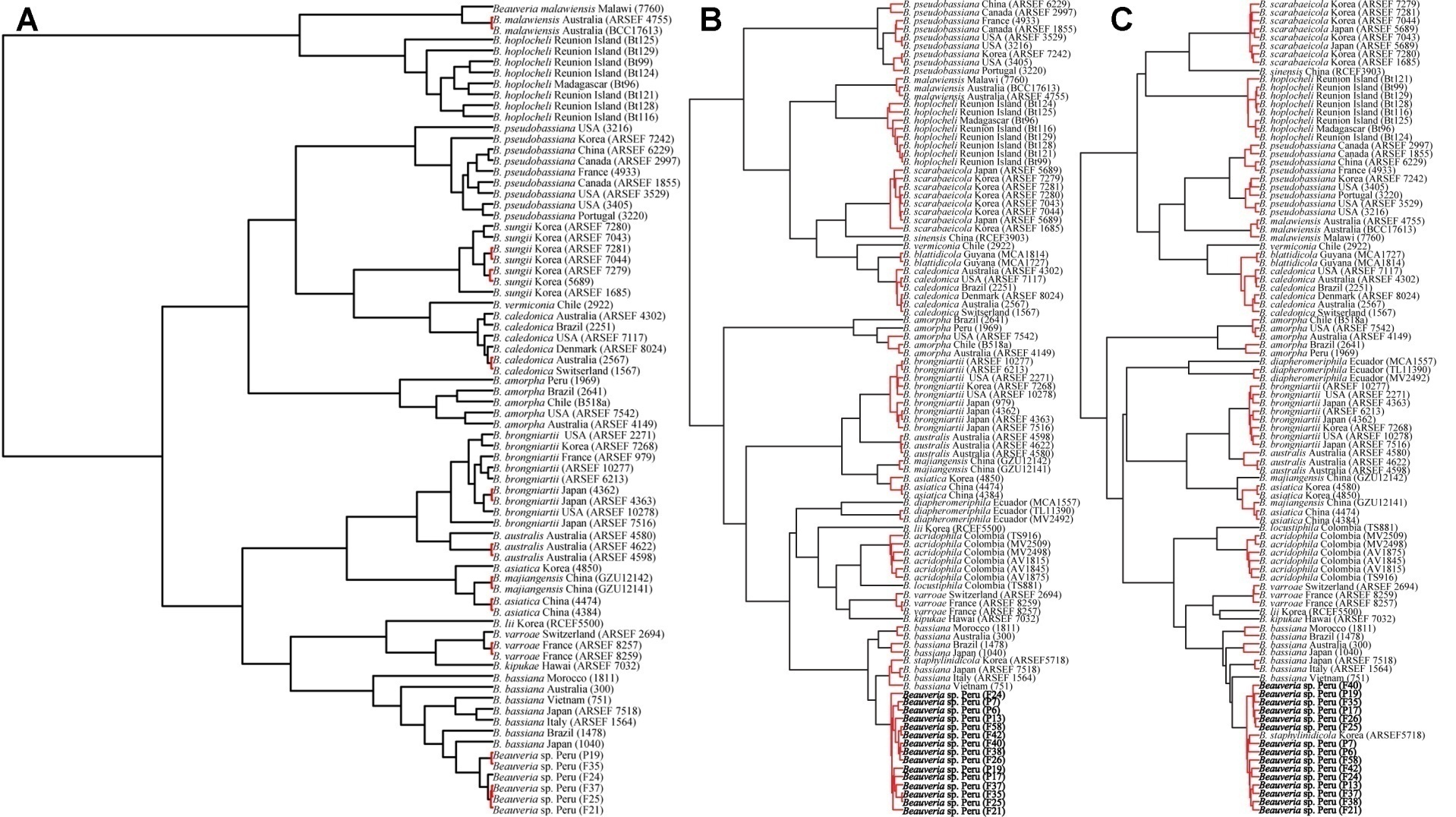
Fig. S4.** Bayesian inference ultrametric gene tree obtained using a Yule tree prior in BEAST with the statistical species delimitation results from GMYC based on *Bloc* (A), *RPB*1 (B), and *Tef*1 (C).
